# Comparing inductive and deductive analysis techniques to understand health service implementation problems: a case study of childhood vaccination barriers

**DOI:** 10.1186/s43058-021-00202-0

**Published:** 2021-09-15

**Authors:** Carissa Bonner, Jane Tuckerman, Jessica Kaufman, Daniel Costa, David N. Durrheim, Lyndal Trevena, Susan Thomas, Margie Danchin

**Affiliations:** 1 Faculty of Medicine and Health, School of Public Health, The University of Sydney, Sydney, Australia; 2 Vaccine Uptake Group, Murdoch Children’s Research Institute, Melbourne, Australia; 3 School of Psychology, The University of Sydney, Sydney, Australia; 4 Pain Management Research Institute, Royal North Shore Hospital, Sydney, Australia; 5 School of Medicine and Public Heatlh, University of Newcastle, Callaghan, Australia; 6 Department of Paediatrics, University of Melbourne, Melbourne, Australia; 7 Department of General Medicine, Royal Childrens Hospital, Melbourne, Australia

**Keywords:** TDF, COM-B, Implementation, Childhood vaccination

## Abstract

**Background:**

Effective implementation requires a comprehensive understanding of individual, organisational and system determinants. This study aimed to compare inductive and deductive analysis techniques to understand a complex implementation issue. We used childhood vaccination as a case study, an issue with wide-ranging barriers contributing to low-vaccine uptake internationally.

**Methods:**

The study is based on the Behaviour Change Wheel framework, which was derived from several levels of theory: the 3 components of the COM-B framework (capability, opportunity and motivation) can be mapped to the 14 domains of the Theoretical Domains Framework (TDF), which is based on 84 underlying constructs. We first conducted a review of systematic reviews of parent-level barriers to childhood vaccination. Subsequently we (1) inductively coded these barriers into a data-driven framework, using thematic analysis, and (2) deductively mapped the barriers to COM-B and TDF domains and constructs. These processes were undertaken by two authors independently, and discrepancies were resolved through discussion. Inductive and deductive results were compared.

**Results:**

The inductive process coded 583 descriptions of barriers identified from the literature into a framework of 74 barriers in 7 categories. The initial definitions used to map the barriers to deductive domains/constructs led to 89% agreement at the domain level. Resolving discrepancies required further definitions at the construct level. Of the 14 TDF domains, 10 were clearly identified in the data from the barrier reviews. Some domains were not specific enough to differentiate between types of barriers (e.g. Environmental Context and Resources), while other domains were not represented in the review data (e.g. Behavioural Regulation).

**Conclusions:**

Using both inductive and deductive analysis techniques can help achieve a more comprehensive understanding of barriers to health service implementation. The inductive categories represented the review data in a clearer way than the theoretical domains, with better differentiation; but the missing deductive domains were useful as a way to identify additional issues to investigate further. Both analysis techniques resulted in a comprehensive list of barriers to vaccination that would not have been achieved using either approach alone. We recommend a hybrid approach combining TDF with broader frameworks, for future researchers conducting evidence syntheses.

Contributions to the literature

- Deductive theoretical analysis techniques to understand implementation problems, such as the TDF and COM-B, may raise different issues compared to inductive data-driven analysis techniques
- This paper describes a process for comparing inductive and deductive analysis techniques to understand an implementation challenge of global significance
- We describe an analysis process using several levels of framework development (84 constructs underlying the 14 TDF domains, which link to the 3 COM-B components) and identify new directions to improve the specificity of theoretical behavioural constructs in future research
- The paper illustrates how inductive and deductive analysis techniques synergise to produce a more comprehensive understanding of health service barriers than using either approach alone

## Background

Effective implementation of a health service programme, guideline or treatment requires understanding a wide range of system, organisational and individual determinants of uptake [1]. This may involve reviewing existing literature for well-established problems or conducting original research if the issue is new. Incorporating theoretical frameworks can ensure all possible drivers are considered [2].

The use of theoretical frameworks enables an understanding of the mechanisms of change from individual to system levels, which can then be targeted in interventions. Multiple theories are used in healthcare, from simple models of individual health behaviour change like the Theory of Planned Behaviour [3], to broader systems thinking approaches to map the complexity of policy drivers [4]. The Behaviour Change Wheel (BCW) is one approach that attempts to bring individual and system level factors together [5], based on the COM-B (capability, opportunity, motivation—behaviour) framework that synthesises 14 behavioural constructs in the Theoretical Domains Framework (TDF) [6] into broader categories.

The TDF summarises the many overlapping constructs in the behaviour change literature and was developed through expert consensus from 128 theoretical constructs in 33 theoretical models of behaviour [7]. It provides an overview of 14 key theoretical constructs that explain health behaviour and is a descriptive framework rather than a theory of causality. A separate systematic review of 19 frameworks for behaviour change interventions led to the BCW, which aims to guide the development of interventions by connecting the determinants of behaviour with behaviour change techniques [5]. Developed in conjunction with the BCW, and at its central core, is the COM-B framework which proposes that behaviour is a product of the interaction between capability (psychological or physical), opportunity (social or physical) and motivation (automatic or reflective) [5, 7].

The COM-B and TDF have been mapped to each other, but there is some duplication of the current 14 TDF domains across the COM-B components. Table 1 summarises this theoretical relationship.

**Table 1:** Relationships between the TDF and COM-B (adapted from Tables 2 and 3 in Cane et al.) [6]

COM-B components	TDF domain definitions
**Capability:** Psychological	**Knowledge:** An awareness of the existence of something
	**Behavioural Regulation:** Anything aimed at managing or changing objectively observed or measured actions
**Capability:** Psychological and physical	**Skills:** An ability or proficiency acquired through practice
**Capability:** Physical	**Memory, Attention and Decision Processes:** The ability to retain information, focus selectively on aspects of the environment and choose between two or more alternatives
**Opportunity:** Physical	**Environmental Context and Resources:** Any circumstance of a person's situation or environment that discourages or encourages the development of skills and abilities, independence, social competence and adaptive behaviour
**Opportunity**: Social	**Social influences:** Those interpersonal processes that can cause individuals to change their thoughts, feelings or behaviours
**Motivation:** Reflective	**Beliefs about Consequences:** Acceptance of the truth, reality or validity about outcomes of a behaviour in a given situation
	**Beliefs about Capabilities:** Acceptance of the truth, reality or validity about an ability, talent or facility that a person can put to constructive use
	**Intentions:** A conscious decision to perform a behaviour or a resolve to act in a certain way
	**Goals:** Mental representations of outcomes or end states that an individual wants to achieve
**Motivation:** Reflective and automatic	**Social/Professional Role and Identity:** A coherent set of behaviours and displayed personal qualities of an individual in a social or work setting
	**Optimism:** The confidence that things will happen for the best or that desired goals will be attained
**Motivation:** Automatic	**Reinforcement:** Increasing the probability of a response by arranging a dependent relationship, or contingency, between the response and a given stimulus
	**Emotion:** A complex reaction pattern, involving experiential, behavioural, and physiological elements, by which the individual attempts to deal with a personally significant matter or event

Primary research is often used to identify barriers to implementation in different health service contexts, and this is the approach generally used with the TDF [7]. Some issues have been well researched, but this evidence must be synthesised in order to inform comprehensive intervention design [8]. Previous reviews have applied theoretical frameworks to help with this. For example, the BCW can be used to describe interventions in terms of broader functions [9], and the COM-B can be used to display barriers and facilitators at multiple levels (patient, provider, system) [9]. The TDF can be used together with the COM-B to group barriers and facilitators of health outcomes [10], or as a stand alone framework [11].

A deductive analysis technique using theory-driven constructs may identify different implementation issues compared to inductive techniques that are data-driven. A deductive application of theory ensures that all psychological constructs relevant to behaviour are considered, even if research has not identified every construct. However, since these theoretical frameworks are based heavily on psychological theory, the internal ‘motivation’ aspect is more clearly defined than the more external ‘opportunity’ aspect. This imbalance does not necessarily align with the prevalence and significance of practical issues in health service implementation, which might be defined as ‘physical opportunity’. A hybrid approach can be used to address this [12, 13], but the extra time and expertise required need to be weighed against the potential benefits.

The aim of this paper is to compare inductive and deductive analysis techniques applied to the same implementation issue and illustrate how these processes can complement each other. We use parent uptake of childhood vaccination as an example of an international issue with wide ranging barriers identified in multiple reviews.

## Method

### Theoretical approach

The study was based on the BCW framework because it incorporates both individual and system level barriers to behaviour and is based on several levels of theory: the 3 components of the COM-B framework can be mapped to the 14 domains of the TDF, which is based on 84 underlying constructs [5].

### Context: The Vaccine Barriers Assessment Tool (VBAT) project

This analysis is based on data gathered for the Vaccine Barriers Assessment Tool (VBAT) project, which aims to design and validate a survey tool to diagnose the causes of under-vaccination in children under 5 years. Developed in Australia and New Zealand, VBAT aims to incorporate both access and acceptance barriers in a comprehensive tool which will include both short and long form versions, for different uses. An overview of systematic reviews of primary studies on barriers to childhood vaccination was conducted, and 583 descriptions of parental barriers to childhood vaccination uptake were extracted and inductively grouped into categories [14]. Barriers were extracted if they were reported from or relevant to the specific perspective of parents of children under 5 years; barriers from the perspective of health professionals or the health system alone were not included. The findings of the review were thematically organised into a framework of barriers. In a separate deductive process, the 583 barrier descriptions were mapped to the 14 domain version of the TDF, to check whether any theoretical determinants of childhood vaccine uptake were missing in the systematic review data. The purpose of this exercise for the VBAT project was to ensure that a comprehensive pool of potential survey questions could be generated that captured both access and psychological or acceptance barriers. The inductive review and development of the VBAT items will be reported separately (manuscript in preparation [15]). In the results of this article, we describe the utility of using both inductive inductive and deductive analysis techniques to identifying drivers of vaccination. Specific terms are used as outlined in Table 2.

**Table 2:** Term definitions and examples for inductive vs deductive analysis techniques

Level of categorisation	Inductive terms	Deductive terms
**Low level (specific)**	*Barrier descriptions* refers to the 583 individual descriptions of implementation issues extracted from systematic reviews in the VBAT review of the vaccination uptake literature	*Construct* refers to the more detailed list of 84 unique theoretical concepts that informed the TDF and COM-B frameworks
***Example***	*Belief that the vaccine is more dangerous than the illness*	*Consequents*
**Mid level**	*Barrier* refers to the 74 groups of barrier descriptions across studies identified in the VBAT review of the vaccination uptake literature	*Domain* refers to the 14 broad categories of behavioural drivers described in the most recent version of the TDF (Theoretical Domains Framework)
***Example***	*Concern about vaccine safety*	*Beliefs about consequences*
**High level (broad)**	*Category* refers to the 7 groups of barriers identified in the VBAT review of the vaccination uptake literature	*Component* refers to the 6 components in the simplest theory-driven framework of behavioural drivers, the COM-B (Capability, Opportunity, Motivation – Behaviour)
***Example***	*Concerns and beliefs*	*Reflective motivation*

### Process

**Fig. 1:**
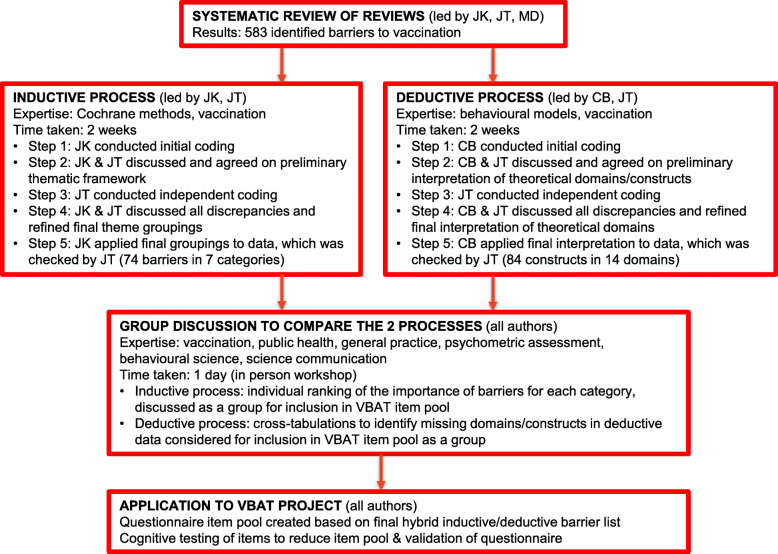
Inductive and deductive processes

Figure 1 illustrates the inductive and deductive processes, supported by regular group meetings with all authors to discuss each step. We used the perspective of *parents* (not health professionals or health systems), which affected the way the deductive categories were applied. The prevalence of domains was examined to determine missing theoretical constructs in the data.

## Results

### Mapping inductive barriers to deductive domains

The initial definitions used to compare inductive barriers with theoretical domains/constructs led to 89% agreement at the domain level. For example, we specified that all barriers relating to the clinic setting will be under the domain of Environmental Context and Resources. Resolving disagreements for the domains and subsequent constructs required further definitions at the construct level before 100% agreement was reached. Table 3 illustrates this for the domain of Environmental Context and Resources, where we decided that issues relating to how appointment times are managed will be under the construct of Organisational culture/climate, while issues relating to inconvenient access for the parent will be under the construct of Person x Environment Interaction. The full list of definitions in available in Appendix.

**Table 3:** Example of definitions required to code TDF domains

Domain	Construct	Notes on decision making
Environmental Context and Resources (any circumstance of a person’s situation or environment that discourages or encourages the development of skills and abilities, independence, social competence and adaptive behaviour)	Environmental stressors	Role of media
	Resources/material resources	Cost issues, lack of supply
	Organisational culture/climate	How clinic is managed (e.g. appointment time)
	Salient events/critical incidents	Specific adverse event/illness in past
	Person x Environment Interaction	Inconvenience to specific parent (e.g. location)
	Barriers and facilitators	General access factors/catch all for ‘other’

Note: there were 14 domains and 84 constructs. Coders CB and JT made notes on their decisions about how the construct was to be applied ot the data, with examples above and full notes in Appendix

**Fig. 2:**
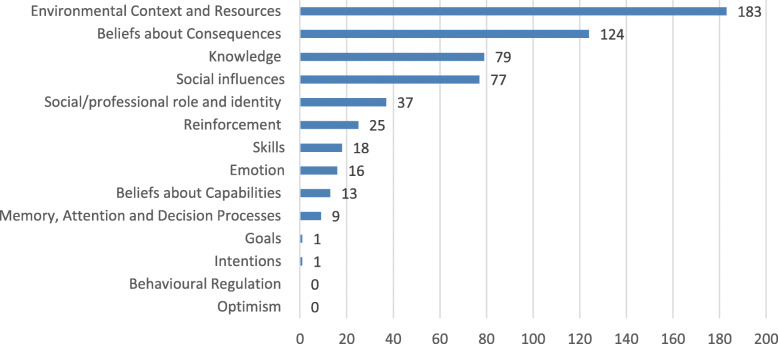
Number of barriers in each TDF domain from inductive data-driven process

Figure 2 shows the number of barriers represented in each theoretical domain. Table 4 shows the relationship between deductive COM-B components and TDF domains, and inductive barriers identified in systematic reviews of primary research. Of the 14 TDF domains, 10 were definitively present in inductive data while 4 domains were not covered in the initial coding: Optimism, Intentions, Goals and Behavioural Regulation (with the exception of two very general barriers for Intentions and Goals with no further explanation). Two domains grouped many different concepts under generic terms (Beliefs within Beliefs about Consequences, Barriers and Facilitators within Environmental Context/Resources). Of the 84 constructs within the 14 TDF domains, many were not identified in the inductive data. This is shown in yellow in Appendix.

**Table 4:** Relationship between inductive and deductive concepts

COM-B component	Deductive concepts (TDF domains)	Inductive concepts (data-driven barriers)
Capability	Knowledge	Lack of information about vaccination, false contraindications
	Skills	Staff are unpleasant or poor communication, language barriers
	Memory, Attention and Decision Processes	Reminder notice, missed opportunities, forgot
	*Behavioural Regulation	**Not represented**
Opportunity	Environmental Context and Resources	First child, low income, media, distance, supply, cost, time
	Social influences	Social exclusion, peer influence, trust, compliance, natural immunity
Motivation	Social/professional role and identity	Traditional beliefs and customs, role of parent, lack of coordinated care
	Beliefs about Capabilities	Can control pathogens child is exposed to, lower parental satisfaction with care
	*Optimism	**Not represented**
	Beliefs about Consequences	Anticipated guilt, vaccine efficacy, disease severity/susceptibility, pain
	Reinforcement	Well-baby clinic counselling, benefit to others, vaccination delay at 3 months
	*Intentions	Practices about health and prevention (*n*=1 with lenient interpretation)
	*Goals	Lack of motivation (*n*=1 with lenient interpretation)
	Emotion	Anxiety about vaccination, fear of needles, psychosocial distress

*Note: These 4 domains were not included in the first round of coding. Intentions and goals were later included after discussion with a very lenient interpretation of the inductive barriers to maximise the number of domains covered, given the aim of the exercise was to generate questionnaire items covering all possible behavioural influences. No inductive barriers could be interpreted as behavioural regulation or optimism

## Discussion

Overall, we found it useful to synthesise health service implementation barriers using both inductive and deductive analysis techniques to gain a comprehensive understanding of the barriers to childhood vaccination. The inductive data-driven categories represented the primary research data in a clearer way than the deductive theoretical domains, with better differentiation; but the four missing theoretical domains were useful as a way to identify key gaps to be addressed in the item pool for developing a new tool to diagnose the causes of childhood under-vaccination.

Resolving conflicts at the domain level was relatively straighforward, with 100% agreement reached quickly. However, there were some barriers that could have been placed in several domains. For example, previous experience of vaccine side effects could be framed as knowledge, beliefs or salient events. Resolving conflicts at the construct level was more difficult because many constructs within a domain were very similar when applied to the brief barrier descriptions extracted from reviews, for example the influence of family member opinions could fit within group identity, social norm or social pressure. The decisions made at construct level were arguably more subjective than the domain level, but both needed to be considered to make sense of many barriers that could be framed in different ways.

For this study, it was necessary to go into more theoretical detail than the commonly used frameworks: the COM-B and TDF. Importantly, the gaps identified in our inductive review would not have been found if the analysis had only been done at the COM-B level, as all six components were addressed by the 10 inductive barrier categories. In addition, the 14 TDF domains were still not specific enough for two coders to reliably map the barrier data so we were required to go back a step to the 84 theoretical constructs that informed the TDF development. We found it helpful to use a combination of domain and construct level to map the data. A previous review using the TDF identified some issues that could not be mapped to the TDF, including clinician and patient characteristics. However, some of these could be mapped at the construct level depending on the framing, such as under professional identity, skills, environment x person and resources constructs [16].

### Practical implications

This paper provides analysis techniques for anyone seeking to understand an implementation issue that already has a large amount of qualitative and/or quantitative research—complementing an earlier paper that focuses on how to apply the TDF in primary qualitative research [7]. There are several practical implications for other researchers seeking to comprehensively understand implementation barriers using theoretical frameworks in this way. Firstly, researchers need to decide on very specific framing for a health situation. In our case, we decided we would only consider the parent perspective on vaccinating their child, which determined how we framed barriers relating to the doctors’ knowledge. Conducting this process from the health professional perspective would produce different results in terms of the theoretical constructs identified in the literature. We included both barriers to the intervention and barriers to implementation but other projects may need to distinguish between these. Secondly, the COM-B framework was not specific enough with uneven explanation of different barrier types, so researchers may need to go into more detail at domain and construct level to interpret the data. Thirdly, theory was useful for identifying gaps in an inductive review of literature, but inductive categories made more sense for the specific implementation topic. The value of using deductive theory-driven analysis techniques may depend on available resources, given this process took 2 authors with prior knowledge of behavioural frameworks around 2 weeks for coding and discussion. For our purposes, this review will inform the development of a diagnostic tool to measure the causes of under-vaccination, requiring us to include the widest possible range of behavioural drivers. For other projects, it may be more prudent to focus only on the theoretical drivers that are within an organisation’s control to address or to identify inductive issues from the perspective of key stakeholders to ensure their interest and support. Future questionnaire developers may benefit from reviewing existing validated survey items prior to a literature review, so that barriers can be linked to established items at the same time.

### Theoretical implications

More generally, this study has implications for theoretical frameworks commonly used in implementation science. Some constructs are vague and became catch alls, such as barriers and facilitators. Others are too specific and hard to distinguish, particularly group vs social norms, which could be combined into one category. In our experience, the decision was often between constructs in different domains, rather than constructs within a domain, suggesting that there are some issues with the way the TDF domains are differentiated. On the other hand, the construct level was often too subjective and detailed to identify clear gaps in data. This suggests that overarching frameworks like the COM-B and TDF need to be supplemented with more context-specific frameworks for different health areas (e.g. prevention versus treatment of infectious disease), targets of behaviour change (e.g. parents versus doctors), and the context (e.g. higher resource settings where psychological barriers may be more important, versus lower resource settings where practical access issues require greater differentiation). Another option would be to use broad implementation frameworks that include practical issues like cost, such as the Consolidated Framework for Implementation Research (CFIR) [17]. Other researchers have found it helpful to combine the TDF and CFIR for a more comprehensive approach [1]. A third option would be to add more specific domains to the next version of the TDF to better differentiate between issues relating to ‘Environmental Context and Resources’. In our review, this covered a very wide range of issues: socio-economic issues such as having low income, societal issues like the influence of media, health system issues like vaccine supply and cost, and individual access issues like distance and time. This was found to be a catch all category in many previous reviews of clinicians and patients using the TDF [16, 18–22], so is not limited to the issue of vaccination barriers. For example, a review of barriers to low back pain guidelines found this domain was common to 4/5 clinician behaviours while many other domains were not covered at all [20]. Another review on diabetic screening identified 17 barriers in this domain versus 6 for the next most common domain [18]. Further development of this construct may need to be specific to different health topics.

For the purpose of the VBAT study, we aimed to identify the widest possible range of behavioural barriers documented in the literature, not the relationships between them, so a framework approach was appropriate. We framed all concepts as ‘barriers’ by reversing concepts framed as facilitators where required, for consistency. VBAT will be used to identify the presence of key access and/or acceptance barriers in specific populations. Once identified, the key barriers would require more specific models or theories to guide intervention development, which may frame the same construct as either facilitator or barrier.

### Strengths and limitations

This study involved independent coding for both inductive and deductive analysis techniques. Our team included a wide variety of expertise to help contextual framing for theoretical constructs as applied to inductive barriers. The limitations include restricting our review data to parent barriers only, which affected the way that health professionals’ and heatlh system barriers were conceptualised. We also applied only one overarching framework based on behaviour change models and acknowledge that there are many other approaches to this theoretical issue.

In conclusion, using both inductive and deductive analysis techniques can help achieve a more comprehensive understanding of health service implementation problems, but the TDF approach needs to be refined in the context of vaccination. We recommend a hybrid approach combining TDF with frameworks such as CFIR, for future researchers conducting evidence syntheses using a theoretical approach. The process is subjective so requires a wide range of expertise to reduce biased interpretation and to maximise utility of the identified barriers for the specified purpose.

## Figures and Tables

**Table 5:**
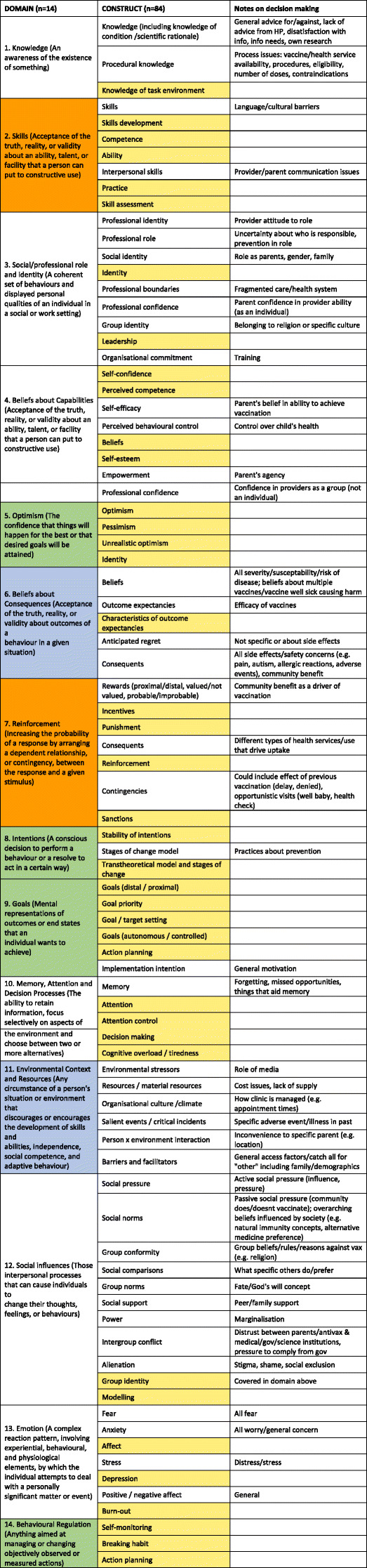
Decision making notes by domain and construct (green/yellow = not represented in data; orange = only included after discussion; blue = ‘catch all’ domains that didn’t differentiate well)

## Data Availability

Data available on request from Carissa Bonner (carissa.bonner@sydney.edu.au).

## Abbreviations

BCW: Behaviour Change Wheel
TDF: Theoretical Domains Framework
COM-B Model: Capability-Opportunity-Motivation-Behaviour Model
VBAT: Vaccine Barriers Assessment Tool
CFIR: Consolidated Framework for Implementation Research
